# Recent Developments and Challenges in Solid-Contact Ion-Selective Electrodes

**DOI:** 10.3390/s24134289

**Published:** 2024-07-01

**Authors:** Lili Gao, Ye Tian, Wenyue Gao, Guobao Xu

**Affiliations:** 1 School of Materials Science and Engineering, Shenyang Jianzhu University, Shenyang 110168, China; liligao@sjzu.edu.cn; 2 State Key Laboratory of Electroanalytical Chemistry, Changchun Institute of Applied Chemistry, Chinese Academy of Sciences, Changchun 130022, China; 3 Shandong Provincial Center for In-Situ Marine Sensors, Institute of Marine Science and Technology, Shandong University, Qingdao 266237, China; gaowy@email.sdu.edu.cn; 4 School of Applied Chemistry and Engineering, University of Science and Technology of China, Hefei 230026, China

**Keywords:** solid-contact ion-selective electrodes, ion-selective membranes, potentiometry, review

## Abstract

Solid-contact ion-selective electrodes (SC-ISEs) have the advantages of easy miniaturization, even chip integration, easy carrying, strong stability, and more favorable detection in complex environments. They have been widely used in conjunction with portable, wearable, and intelligent detection devices, as well as in on-site analysis and timely monitoring in the fields of environment, industry, and medicine. This article provides a comprehensive review of the composition of sensors based on redox capacitive and double-layer capacitive SC-ISEs, as well as the ion–electron transduction mechanisms in the solid-contact (SC) layer, particularly focusing on strategies proposed in the past three years (since 2021) for optimizing the performance of SC-ISEs. These strategies include the construction of ion-selective membranes, SC layer, and conductive substrates. Finally, the future research direction and possibilities in this field are discussed and prospected.

## 1. Introduction

The 21st century is an era of great health, and human beings are constantly pursuing complete health in psychology, physiology, society, and environment. Green ecological environment and health have become a common aspiration of all humankind. Environmental pollution and life health have always been issues of great concern. Therefore, the development of sensors that can achieve timely monitoring and on-site analysis of target substances is of great significance for people to create a green ecological environment and understand their own health status. With the development of technology, various electrochemical sensors have been studied, including ion-selective electrodes, which can quantify the concentration of target ions and relate it to electrical output parameters, such as potential, impedance, and current, and have become the most promising biological and chemical sensing devices due to their excellent detection performance, low cost, low energy consumption, simple equipment, and single output parameters [1]. It has been found that traditional liquid-contact ion-selective electrodes (LC-ISEs) have inherent limitations [2]: (1) The evaporation, permeation, sample temperature, and pressure changes of the inner filling solution affect the electrode response; (2) Osmotic pressure resulting from the difference in ion strength between the sample and the inner filling solution can cause water to enter and leave the internal filling solution, resulting in large volume changes or stratification of the ISM; hence, LC-ISEs must be used and maintained carefully, which incurs high cost; (3) There is a steady-state ionic flux between the inner filling solution and the test solution; (4) It is difficult to reduce the volume of the inner filling solution to the milliliter level, making it difficult to miniaturize the electrode. These factors have hindered the development of LC-ISEs in the past. In recent years, SC-ISEs have been continuously developed and applied. Compared with LC-ISEs, a solid contact (SC) layer is formed between the ion-selective membrane (ISM) and the electronic conduction substrate (ECS) in the structure of SC-ISEs to replace the liquid contact, serving as an ion–electron conversion layer, thereby eliminating the cumbersome liquid-phase contact and completely solving the problem of the need for an internal filling solution in LC-ISEs. Combining SC-ISEs with potentiometric analysis, the application performance of SC-ISEs is significantly improved due to the inherent advantage of potentiometric analysis in terms of power efficiency. In an electrochemical process, when a negligible bias current flows, the potential at the interface between the working electrode and the reference electrode can be accurately measured [3,4]. Compared with voltammetric and amperometric sensors, this technique is less affected by Ohm’s drop and interference effects [5]. In addition, previous studies have demonstrated that electrodes based on potentiometric analysis can achieve a miniaturized design without sacrificing sensitivity [6]. Therefore, SC-ISEs have the advantages of easy miniaturization, even chip integration, easy carrying, strong stability, and more favorable detection in complex environments. They have been widely used in conjunction with portable, wearable, and intelligent detection devices, as well as in on-site analysis and timely monitoring in the fields of environmental monitoring, food safety, and medicine.

Although SC-ISEs have developed rapidly in recent decades, there are still many issues such as poor stability, low reproducibility, and high toxicity of ion carriers in wearable devices. Previously, there have been some related review papers on SC-ISEs, but the publication years are mostly before 2021 [7,8]. In order to address these issues and help readers from a wide range of disciplines to conduct more in-depth research in the field of SC-ISEs, this article provides a comprehensive review of the composition of sensors based on redox capacitive and double-layer capacitive SC-ISEs, as well as the ion–electron transduction mechanisms in the solid-contact (SC) layer, particularly focusing on strategies proposed in the past three years (since 2021) for optimizing the performance of SC-ISEs. These strategies include the construction of ion-selective membranes (SIMs), the SC layer, and conductive substrates. Future research directions and possibilities in this field are also discussed and prospected.

## 2. Composition and Working Principle of Solid-State Potential Sensors

There are two design schemes for solid-state potential sensors. One is the standard configuration, which consists of an SC-ISE and a solid-state reference electrode (RE) in a dual electrode layout [9]. The other configuration is the three-electrode design, which includes an additional counter electrode (CE) compared to the standard configuration [10]. Among them, SC-ISEs are composed of the ISM, the SC layer, and the ECS [11]. The ISM is the most important component of SC-ISEs and has been widely studied in the past few decades. The ISM is composed of an ion carrier, an ion exchanger, a plasticizer, and a polymer matrix. These components have different functions. The ion carrier is responsible for selectively extracting the target ions from the sample interface into the ISM. Therefore, the ion carrier generally has a functional group structure that can accommodate a large number of target ions or a functional group structure with coordination space and coordination sites that can fulfill ion-specific binding. In addition, highly hydrophobic ion carriers can also prevent the leakage of membrane components into the sample [12]. Currently, a large number of natural or synthetic ion carriers, mainly cationic uncharged carriers or anionic charged carriers, have been developed [13]. The ion exchanger is an indispensable and important part of the ISM. It can introduce a large number of ions with opposite charges into the membrane to reduce interference. It can also facilitate the exchange process between the ISM and target ions and increase the conductivity of the ISM. This characteristic is very favorable for the miniaturization of SC-ISEs. When the ion carrier is electrically neutral, using an ion exchanger with an opposite charge to the target ions can ensure that the ISM has a considerable number of ion sites with opposite charges, thereby achieving the “Donnan exclusion effect” for electrically neutral carriers [14]. Currently, commonly used cationic ion exchangers include sodium tetrakis (pentafluorophenyl) borate (NaTFPB), potassium tetrakis (4-chlorophenyl) borate (KTPCIPB), and potassium tetrakis [3,5-bis (trifluoromethyl) phenyl] borate (KTFPB). The polymer matrix provides the necessary physical and mechanical properties for the ISM and serves as the backbone of the ISM. Currently, widely used polymer matrices include polyvinyl chloride (PVC) and its derivatives, acrylic esters, polyurethane, polystyrene, and silicone rubber. The plasticizer is used to improve the plasticity or fluidity of the active components in the ISM. Selecting an appropriate amount of plasticizer can ensure both the physical properties of the ISM and its high fluidity. In addition, plasticizers with different polarities have different dielectric constants and liposolubilities. Therefore, the selection of the plasticizer should meet its compatibility with the ion carrier, thus optimizing the selectivity of the ISM based on the ion carrier [15]. Common plasticizers used in polymer membranes are bis(2-ethylhexyl) sebacate (DOS), dibutyl phthalate (DBP), dioctyl phthalate (DOP), and 2-nitrophenyloctyl ether (NOPE).

**Figure 1:**
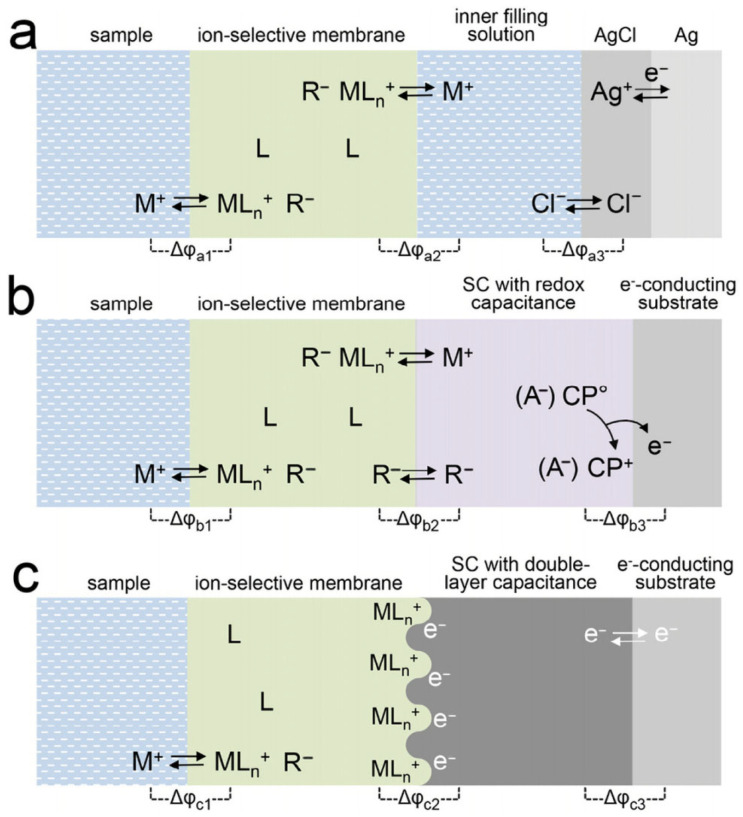
Schematic representation of all relevant interfaces within different types of ISEs with cation (M^+^)-selective membranes that contain an electrically neutral ionophore (L) and anionic sites (R^−^): (**a**) a conventional ISE with an inner filling solution; (**b**) an SC-ISE based on an anion (A^−^, R^−^) doped-conducting polymer (CP) SC with a high redox capacitance; (**c**) an SC-ISE based on a high-surface-area SC exhibiting a high double-layer capacitance [16].

The potential difference between SC-ISEs and the reference electrode (including the connected metal wire) is the measured electromotive force (EMF), and the total EMF is equal to the sum of all interfacial potentials [16]. When the sensor is in operation, the target ions in the sample are specifically recognized by the ion carrier in the ISM, resulting in an input signal [17]. Subsequently, unlike traditional LC-ISEs (Figure 1a) where a reversible redox reaction (Equation (1)) is induced through the internal filling solution through the inner reference electrode (Ag/AgCl), the ions in the ISM as freely movable charge carriers are conducted to the SC, achieving an ion–electron conversion at the ISM/SC interface. Depending on the different conversion mechanisms, SC can be classified into oxidation–reduction capacitance-type and electric double-layer (EDL) capacitance-type.

The oxidation–reduction capacitance-type SC-ISEs introduce a conductive material with a large oxidation–reduction capacitance between the ECS and ISM. Conducting polymers (CPs) not only exhibit electronic conductivity but also demonstrate ionic conductivity through doping, making them an efficient ion–electron transducer with both electronic and ionic conductivities. Similar to the redox reactions of the built-in reference electrode in traditional LS-ISEs, the oxidation–reduction reactions (Figure 1b) occurring during the conversion of charge carriers from ions to electrons in CP-based SC-ISEs can be represented by Equations (2) and (3), and these two reaction steps determine the potential difference (△φ_b3_) at the CP/ECS interface, while the distribution of ions in CPs and the ISM determines the potential difference (△φ_b2_) at the interface between the two phases.

Ag_(S)_ + Cl^−^_(aq)_ ⇌ AgCl(S) + e (1)

CP^+^A^−^_(SC)_ + M^+^_(SIM)_ + e^−^ ⇌ CP°A^−^M^+^_(SC)_ (2)

CP^+^R^−^_(SC)_ + e^−^ ⇌ CP°_(SC)_ + R^−^_(SIM)_ (3)

where CP represents the conducting polymer and A^−^ refers to the doping ions (such as poly(4-styrenesulfonate) polyanion, PSS^−^). M^+^ and R^−^ represent the target ions (such as K^+^) and the hydrophobic counter ions (such as tetraphenylborate derivatives), which can be transferred through the interface between the ISM and CPs. Currently, the deposition of CPs on the ECS is typically achieved through methods such as drop casting from polymer solutions or electrochemical polymerization, which are both conducive to mass production and commercialization of the sensor.

In EDL capacitance-type SC-ISEs, one side of the ISM/SC interface carries ionic charges due to the accumulation of cations and anions from the ion-selective membrane, while the other side carries charges formed by electrons or holes in the SC layer. Therefore, the ion–electron transduction in this type of SC-ISEs can be simply described as the formation of an asymmetric capacitor at the ISM/SC interface (Figure 1c). As the concentration of target ions changes, some electrons are capacitively coupled with the SC, leading to changes in the potential compensation ions of the EDL potential and indirectly calibrating the ion activity [18]. Carbon materials such as carbon nanofibers, carbon nanotubes (CNTs), graphene (GP), and carbon black (CB) exhibit chemical inertness, insensitivity to gases, light, and redox substances; and are widely utilized in sensor technology due to their unique chemical, mechanical, optical, and electrical properties [19]. Their superior conductivity has made them an attractive and promising material for surface conductivity (SC) with a confirmed dependence on the electrolyte-driven layer (EDL) capacitive conduction mechanism [20,21,22,23]. Bene et al. [24] improved the electrochemical detection performance of potential sensors by using nanocomposites prepared by doping double-walled carbon nanotubes (DWCNTs) into PEDOT and PPy. Metal oxides, which are ionic compounds containing positive cations and negative oxygen ions, possess unique optical and electrical characteristics. Copper oxide nanoparticles (CuO) exhibit high conductivity and are p-type semiconductors. Abdelaal et al. [25] developed a novel ion–electron transducer layer by synthesizing nanocomposites incorporating Ag/Cu0 nanoparticles with t-butylcalix[8]arene (BCX8).

Due to the high conductivity of most EDL capacitance materials, Δφ_c3_ is negligible. Unlike the case of solid contact with CPs, the interface potential (Δφ_c2_) between the SC/ISM is neither determined by ion distribution between the two phases nor by the redox reaction. Furthermore, there is no charge transfer at the SC/ISM interface, making φ_c2_ undefined. However, φ_c2_ is related to the charge amount ΔQ in the electric double layer, and its potential change can be described by Equation (4).

Δφ_c2_ = ΔQ/C (4)

where △Q is the charge amount passing through the SC/ISM, and C is the EDL capacitance. Therefore, the larger the EDL capacitance of SC-ISEs based on EDL capacitance materials, the more stable the potential response signal.

In order to improve the performance of SC-ISEs, current research mainly focuses on the development of new materials for each component in the ISM and the adjustment of their content. As the bridge between the ECS and ISM, SC is an important component of SC-ISEs and has been the main focus of research in recent years.

## 3. Index of Characteristics

The International Union of Pure and Applied Chemistry (IUPAC) provides recommendations for evaluating the performance indicators of ISEs, and several important indicators are commonly used to evaluate the sensing performance of SC-ISEs.

(1) Selectivity: The selectivity of SC-ISEs refers to their ability to detect the target ions while disregarding other interfering ions. In practical measurements, SC-ISEs may also respond to some interfering ions, leading to a deviation of the response slope from the theoretical value and a decrease in sensitivity. An ideal SC-ISE has high selectivity, ensuring accurate information about the presence and activity values of target ions. The selectivity coefficient is usually calculated based on the two-solution method in mixed solutions [26]. A smaller selectivity coefficient indicates better selectivity and stronger anti-interference ability of the SC-ISE.
(2) Reproducibility: Over time, the ionized compounds in the membrane structure enter the aqueous solution, and the stability of the electrode gradually decreases, resulting in a decrease in the slope [27]. Currently, SC-ISEs must be periodically calibrated to meet the requirements of practical testing, and the pre-calibration process is essential, which relates to the accuracy and reproducibility of the test results. However, complex or frequent calibration processes consume a significant amount of time and increase costs, especially for commercial potential sensors, which require users to have certain professional knowledge. It has been found that adding oxidable/reducible active substances with buffering capabilities to the SIM can significantly improve the reproducibility of the initial E° of carbon-based all-solid-state ion-selective electrodes [28]. However, the loss of oxidable/reducible active substances caused by the ion exchange in the ISM may lead to a gradual drift of E°. Therefore, achieving high reproducibility of the standard electrode potential remains a challenge and an important step toward achieving calibration-free measurements.
(3) Detection limit and detection range: Both excessively high and low activity of target ions in the test solution can cause the electrode response to deviate from the theoretical value of the Nernst slope. When the concentration of the target ions is too low, interfering ions can enter the selective membrane and interfere with the electrode response, resulting in the limit of detection (LOD). When the activity of the target ions is too high, the ISM will undergo co-extraction with the test solution, resulting in the detection upper limit [29]. According to the definition by IUPAC, the intersection of the extension lines of the horizontal and linear parts represents the activity of the target ions corresponding to the detection upper limit or detection lower limit; the linear part between the detection upper limit and detection lower limit represents the detection range of the electrode.
(4) Response time: The response time refers to the time required for the electrode’s potential to reach 95% of the equilibrium value after immersing from one sample solution to the next. It reflects the speed at which the potential value of SC-ISEs reaches equilibrium.
(5) Lifespan: The lifespan of an electrode refers to the duration of time the electrode can function properly while maintaining its performance indicators. Factors such as aging of the membrane matrix, loss of ion carrier and membrane additives, and external environmental damage to the membrane components can all affect the lifespan of SC-ISEs. Wardak et al. [30] modified polymer membranes with a nanocomposite composed of carbon nanofibers and ionic liquid (1-hexyl-3-methylimidazolium hexafluorophosphate), which significantly enhanced the strength of the ion-selective membrane (ISM), and the electrode retained its properties for four months, with negligible changes in the standard potential (E°) and Nernst slope.
(6) Stability: The stability of the electrode refers to the fluctuation and drift of the electrode’s measured potential during the operation. Ideally, SC should have a non-polarizing interface with a high exchange current density [31]. However, in practical testing, the input current of the measurement amplifier inevitably leads to its charge–discharge, causing polarization of the electrode to varying degrees and resulting in a potential drift. Currently, the stability of the electrode is evaluated using the reverse current timing potential method proposed by the Bobacka group [32].

## 4. Construction Strategies and Applications of SC-ISEs

### 4.1. Oxidation–Reduction-Type SC-ISEs

CPs are considered to be one of the most effective ion–electron transducers. Among the many SC materials studied, poly(3-octylthiophene) (POT), polyaniline (PANI), and poly(3,4-ethylenedioxythiophene) (PEDOT) are the most commonly used.

**Figure 2:**
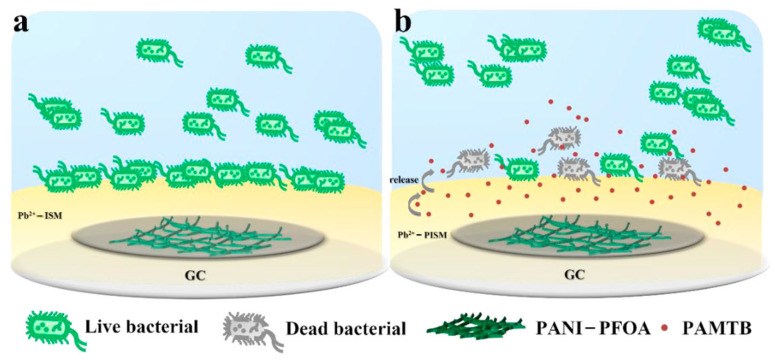
(**a**) Biofouling process on Pb^2+^-ISM and (**b**) antifouling mechanism of Pb^2+^-PISM [38].

In environmental monitoring and water safety analysis, the stability, response time, sensitivity, and lifespan of SC-ISEs are adversely affected when the ISM is covered by biofilm on the electrode surface [33,34]. In recent years, capsaicin and its derivatives have been widely used for their ability to inhibit microbial adhesion [35] in applications such as anti-fouling coatings and antibacterial ultrafiltration membranes [36,37]. Liu et al. [38] successfully prepared a lead ion-selective electrode (GC/PANI-PFOA/Pb^2+^-PISM) based on PANI by incorporating an environmentally friendly capsaicin derivative (propyl 2-(acrylamidomethyl)-3,4,5-trihydroxy benzoate, PAMTB) into the ISM. First, PANI doped with PFOA was electrodeposited on a glassy carbon electrode (GC) to prepare the hydrophobic SC (PANI-PFOA) (Figure 2). Then, a lead ion-selective membrane (Pb^2+^-PISM) composed of lead ion carrier IV, PAMTB, o-NPOE, PVC, and NaTFPB was assembled on the surface of the SC. The long-term anti-fouling effect of the sensor was evaluated, and the study showed that the introduction of PAMTB in the ISM effectively inhibited bacterial attachment on the surface of PISM, thereby improving the overall anti-fouling performance of the SC-ISEs. The excellent antimicrobial performance is attributed to the leakage of PAMTB, which contains abundant phenolic hydroxyl and amide functional groups, from the ISM when it is immersed in the solution. This disrupts the cell membrane structure of Escherichia coli and/or causes osmotic stress, leading to bacterial cell deformation, collapse, and rupture, resulting in bacterial inactivation [39]. In addition, PISM doped with PAMTB exhibits slightly increased hydrophilicity, which is helpful in reducing the adhesion of biofouling. Under optimal conditions, the constructed sensor has no water layer, high selectivity, stability, and an LOD of 1.9 × 10^−7^ M for Pb^2+^ in water, with a response slope of 28.5 ± 0.8 mV/decade and a response time of 20 s. This study provides a new strategy for developing anti-fouling SC-ISEs in complex water systems.

**Figure 3:**
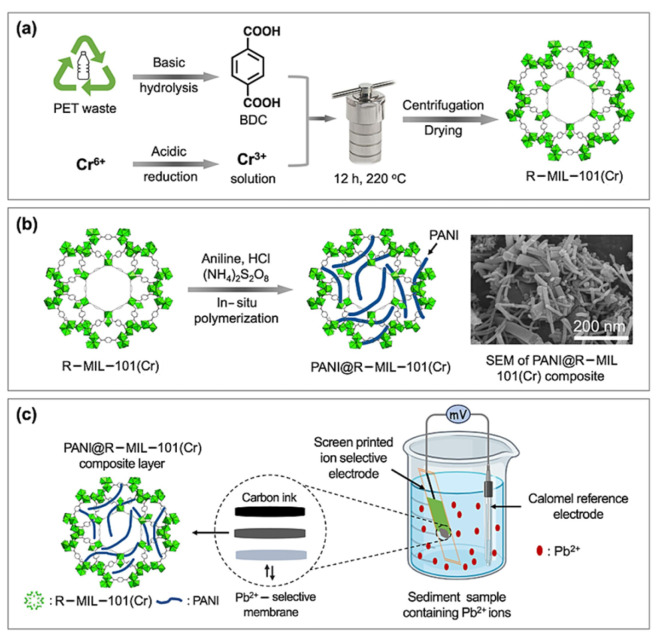
(**a**) R-MIL-101(Cr) synthesis from recyclable PET and Cr^6+^. (**b**) Polymerization of aniline over R-MIL-101(Cr) to produce PANI@R-MIL-101(Cr) composite. (**c**) Potentiometric experiment setup for determining Pb^2+^ ions in the sediment sample [48].

CPs can facilitate a charge transfer at the sensor interface and enhance the potential stability of ISEs [40,41,42]. However, due to certain limitations of CPs, such as sensitivity to light, redox side reactions, and the presence of water layers at CP/ISM interfaces, CP-based SC-ISEs are limited in practical applications [43]. Composite materials of CPs with highly stable materials are considered effective solutions to improve the hydrophobicity and long-term stability of SC-ISEs. Metal–organic frameworks (MOFs) have emerged as promising candidates for improving CP performance due to their unique properties, such as crystal structure, high porosity, and excellent chemical inertness and thermal stability [44]. The MIL-101(Cr) framework possesses a large pore size and surface area, as well as superior chemical inertia and thermal stability [45], and can effectively convert ion signals passing through the ISM into electronic signals between the metal contact and the sensing element [46]. Additionally, the hydrophobic nature of MIL-101(Cr) can prevent the formation of water layers at SC/ISM interfaces and minimize potential drift [47]. Keshta et al. [48] developed a low-cost and HF-free synthetic approach to obtain MIL-101(Cr) (R-MIL-101(Cr)) from organic waste (PET bottles) and inorganic waste (Cr_2_O_7_^−^) (Figure 3). PANI-based R-MIL-101(Cr)-conductive composite material (PANI@R-MIL-101(Cr)) was prepared by an in situ polymerization method, and then PANI@R-MIL-101(Cr) was fixed on the surface of physically polished and DI-washed solid contact screen-printed electrodes (SC-SPEs) using drop casting. Finally, a SIM membrane composed of potassium tetrakis-4-chlorophenyl borate (KTPCB) as a cation exchanger, β-cyclodextrin as an ion carrier, di-n-butyl sebacate (DBS) as a plasticizer, and PVC as a polymer matrix was used to construct the SC-Pb^2+^-ISE for rapid on-site detection of Pb^2+^ in environmental samples. The study demonstrated that PANI@R-MIL-101(Cr) exhibited fast ion–electron transport characteristics with low impedance and high capacitance, while its surface area (87.50 m^2^/g) and hydrophobicity (CA = 62.85°) were higher than pure PANI. These excellent characteristics enhance the sensor’s conductivity for faster ion-to-electron transfer and hinder the formation of water layers at the electrode/SIM interface. Under optimal conditions, the sensor achieved an LOD for Pb^2+^ as low as 1 × 10^−7^ mol/L, a potential drift of 0.9 mV h^−1^, a response time of 5 s, and a lifespan of 70 days. This strategy provides new insights for reducing the production cost of Pb^2+^ sensors and opens the door for the development of new composite materials. However, developing low-cost sensors capable of rapid and highly sensitive detection of other metal ions remains a challenge.

**Figure 4:**
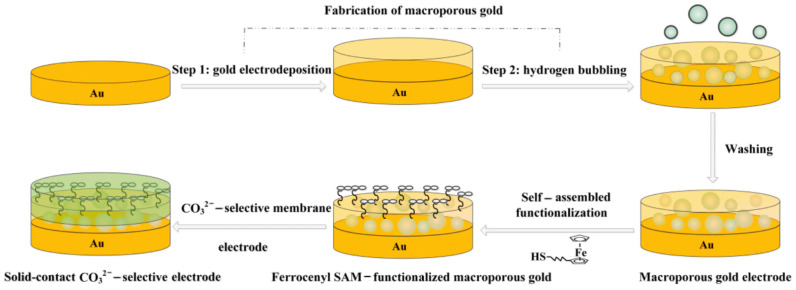
Schematic representation of fabrication of the SC-ISEs based on functionalized ferrocenyself-assembled monolayer [50].

Another potential drawback of using CPs as SC is their continuous redox potential behavior, which presents a challenge for improving the reproducibility of ASS-ISEs with CPs layers. Although the shorting method can improve this drawback, it becomes relatively time-consuming toward the end of the process. In addition to CPs, various other oxidation–reduction SC materials have been developed, such as oxidation–reduction self-assembled monolayers (SAMs) and novel oxidation–reduction pairs. However, SAMs have a limited oxidation–reduction capacity, resulting in less-than-ideal potential reproducibility for SAM-based all-solid-state ISEs (standard deviation of standard potential, E°, ±10 mV) [49]. To address this issue, Mu et al. [50] proposed a strategy to functionalize macroporous gold (m-PG) with a ferrocene-based SAM (FcC_6_SH) and incorporate it into polymeric solid-contact ISEs for carbonate ions (CO_3_^2−^) (Figure 4). The membrane components included carbonate ionophore VII, tridodecylmethylammonium chloride (TDMACl), PVC, and di-n-butyl sebacate (DOA). Firstly, m-PG substrate was prepared on a gold electrode surface using an electrochemical self-template method combining gold electrodeposition and hydrogen bubbling. Then, the FcC_6_SH SAM with thiol groups anchored to the m-PG thin film (Au/m-PG/FcC_6_SH) was formed through gold–sulfur bonds. Finally, the SIM membrane was applied to the Au/m-PG/FcC_6_SH surface. FcC_6_SH not only prevents the formation of water layers at the ECS/SIM interface, but also facilitates charge transfer at the ECS and SIM interface due to its highly electron-rich sandwich structure, resulting in stable changes in the standard potential (E°) of the SC-ISEs (standard deviation of E° of 1.5 mV). Additionally, the exceptional conductivity and large surface area of m-PG with its porous structure were found to enhance the potential performance of the sensor compared to planar gold ECS. Under optimal conditions, the sensor achieved an LOD of 1.1 × 10^−5^ M for CO_3_^2−^, a response slope of 28.3 ± 0.4 mV/decade, improved potential stability, and a lifespan of at least 120 days. This approach, which transformed the electrode substrate from a planar to a porous structure to enhance the redox capability of the SC layer, provides new insights into constructing stable and reliable SC-ISEs.

Nickel–cobalt–sulfide (NiCo_2_S_4_) is a well-known supercapacitor material in the field of energy storage, with a significantly higher redox capacitance (~2000 F g^−1^) than individual metal sulfides [51]. Li et al. [52] utilized a one-step hydrothermal or solvothermal method to form a uniform SC on a glassy carbon (GC) surface, followed by constructing an all-solid-state Ca^2+^ ion-selective electrode (NiCo_2_S_4_-based for all-solid-state solid-contact Ca^2+^-ISE) using calcium ion carrier II (ETH129), NaTFPB, PVC, o-NPOE, and other membrane components. The constructed ASS-Ca^2+^-ISE exhibited high reproducibility (standard deviation of E° of 0.35 mV) and a low potential drift of 1.77 μV s^−1^, especially the significantly improved stability owing to the high redox capacitance of 565 μF. The excellent electrochemical performance was attributed to NiCo_2_S_4_, which provides remarkable redox capacitance through the reversible redox reactions of Ni^3+^/Ni^2+^ and Co^4+^/Co^3+^/Co^2+^ [53]. Under optimal conditions, the sensor achieved an LOD for Ca^2+^ as low as 5.0 × 10^−7^ M, with a Nernst slope of 27.5 ± 0.2 mV/decade. This potential sensor is expected to be used for calibration-free potential measurements.

**Figure 5:**
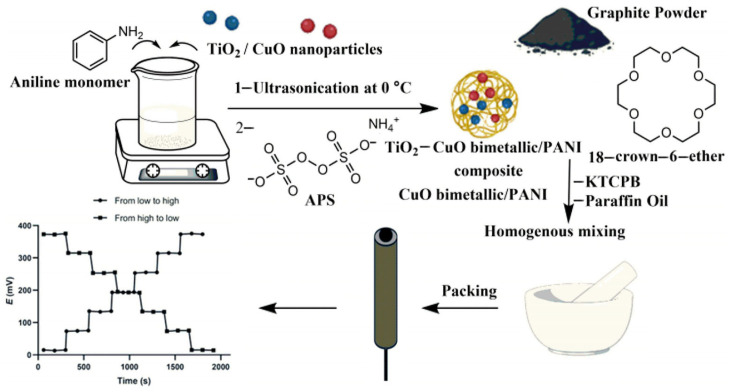
Schematic illustration of the fabrication protocol of the proposed CPE [57].

In recent years, another type of SC-ISE, paste electrodes, has attracted widespread attention in the field of electrochemistry [54,55]. The advantages of this electrode lie in its easy preparation and simple structure; especially after being used, a new electrode can be obtained by peeling off the ISM layer, reapplying a small amount of paste, and re-covering the surface with the ISM. The paste used in paste electrodes should meet certain requirements, such as facilitating rapid ion–electron transduction and having a non-polarized surface between the paste and ISM. Additionally, the paste should be hydrophobic, have low water absorption, and be cost-effective and non-toxic. Moreover, the combination of CPs with single-metal-oxide nanoparticles has been studied for ion–electron transducers, showing that modifying CPs with metal oxide nanoparticles as fillers can improve the electrical performance of CPs [56,57]. Titanium dioxide (TiO_2_) possesses attributes such as high mechanical strength, strong binding energy, high electrical conductivity, excellent chemical stability, large surface area, and non-toxicity. Niemiec et al. [58] modified carbon black (CB) paste based on poly(3-octylthiophene-2,5-diyl) (POT) with hydrated ruthenium dioxide (RuO_2_) to fabricate a novel carbon paste ion-selective electrode for nitrate ion (NO_3_^−^) determination in soil. The high capacitance and low charge transfer resistance of hydrated ruthenium dioxide significantly reduced the potential drift of the sensor. Under optimal conditions, the sensor was unaffected by light and pH conditions ranging from 2 to 10, achieving an LOD of 10^−5.32^ M for NO_3_^−^. Similarly, Abdallah et al. [59] polymerized aniline in the presence of TiO_2_ and CuO nanoparticles to obtain a TiO_2_-CuO bimetallic/PANI nanocomposite, which was used as a transducer in a carbon-paste electrode (Figure 5). An ion-selective carbon-paste electrode for the determination of vildagliptin concentrations in bulk drugs, tablets, and human plasma was developed using 18-crown-6 ether as the ion carrier. The study showed that the presence of TiO_2_ and CuO simultaneously produced a synergistic effect, resulting in better electrical performance of the carbon-paste electrode based on TiO_2_-CuO bimetallic compared to the single-metal/polyaniline nanocomposite electrodes. Under optimal conditions, the electrode achieved an LOD of 4.5 × 10^−9^ M for vildagliptin, a Nernst slope of 60.04 ± 1.4 mV/decade, and a response time of 10 s ± 1.3. Furthermore, the absence of a water layer between the carbon paste and the metal conductor resulted in high stability and a long lifespan (137 days) for the sensor.

**Figure 6:**
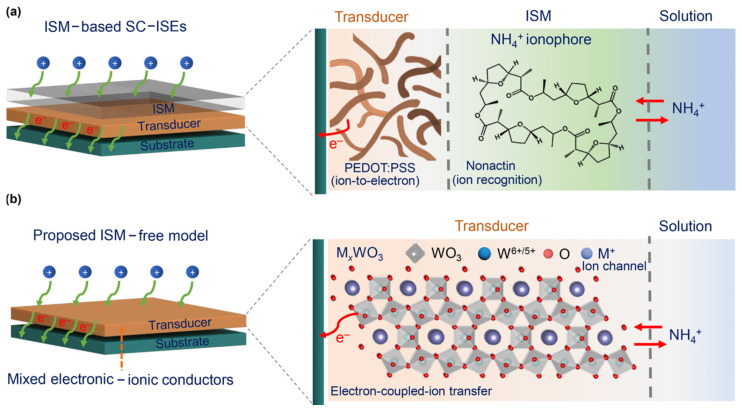
(Color online) Comparison of the state-of-the-art and proposed ISM-free potentiometric ion sensors. (**a**) Present ion sensors based on traditional SC-ISEs with three layers, i.e., substrate, SC transducer, and ionophore-containing ISM. The transducer transforms ion concentration into an electronic signal (e.g., PEDOT:PSS). The ISM plays a role in ion recognition (selectivity) based on organic ionophores (e.g., nonactin ionophore for NH_4_^+^ recognition). (**b**) The proposed ISM-free potentiometric ion sensors with only the substrate and SC transducer of MxWOs. The M_x_WO_3_ works as a bifunctional layer with ion-to-electron transduction through the redox reaction of W^6+/5+^ (electronic conductor) and ion recognition through ion transport in the lattice (ionic conductor) [62].

The construction strategy of the ISM in SC-ISEs has always been one of the important factors in research. On the one hand, the leakage of ISM components into the solution can lead to the degradation or failure of SC-ISEs and contamination of the sample. It is noteworthy that the ion carriers in the SIM components are not only expensive but also toxic [60], which may affect human health during the preparation and use of SC-ISEs, especially for wearable applications. In this regard, Joon et al. [61] coated a layer of silicone rubber (SR) on the surface of a PVC-based ISM to prevent the leakage of membrane components such as ion carriers into the solution. Interestingly, the SR layer not only did not affect the selectivity of SC-ISEs, but also improved the reproducibility of the electrode. On the other hand, the hydration effect at the interface between SC and the ISM can cause the potential shift of SC-ISEs. Although increasing the hydrophobicity of SC is an effective measure, the hydrophilic phenomenon of the ISM is unavoidable. Therefore, the transport of water molecules across the membrane cannot be completely inhibited. Moreover, in long-term analysis and monitoring, SIMs are generally fragile, and mechanical collisions in special detection environments can cause irreversible damage to the ISM. In response to this, Tang et al. [62] proposed a constructive strategy. They selected M_x_WO_3_ (M = Na, K) as a dual-functional material for ion recognition and ion–electron transduction. NaWO_3_ and K_0.3_WO_3_ were anchored on the surface of a Au electrode with polyethylene terephthalate (PET) as the substrate to construct pH and NH_4_^+^ potentiometric sensors, along with a Ag/AgCl solid-state RE, forming a wearable ion-selective sensor array without an ISM (wearable bioelectronic ion-sensing array) (Figure 6). WO_3_ originally has a monoclinic crystal structure, and its distorted crystal structure hinders ion diffusion and electron transfer [63]. However, the group chose NaWO_3_ and K_0.3_WO_3_ due to the incorporation of potassium and sodium, which makes the WO_3_ crystal structure more ordered and symmetrical. In the crystal structure, [WO_6_] forms large channel centers in a square shape (NaWO_3_) and hexagonal shape (K_0.3_WO_3_), enabling the free movement of Na^+^ and K^+^ in these channels. Meanwhile, these channels provide electrons for the WO_3_ lattice, and the ion–electron transduction is achieved through the oxidation–reduction of W^6+/5+^. Previous studies have shown that the ionic radius determines the transport capacity of the channels for ions (H^+^ < K^+^, NH_4_^+^ < Na^+^ < Ca^2+^) [64]. It is noteworthy that due to the very close ionic radii of K^+^ and NH_4_^+^, the anti-interference ability of the SC-ISEs to K^+^ is particularly important. Compared to K^+^, the working electrode of K_0.3_WO_3_ has a lower replacement energy for NH_4_^+^, thus showing high selectivity for NH_4_^+^ (rather than K^+^). In addition, the working electrode of NaWO_3_ in water can be divided into two layers: one is the hydrated HxWO_3_ layer and the NaWO_3_-SC layer formed by the replacement of surface NaWO_3_ with protons, and the potential difference at the HWO_3_/solution interface is similar to that at the proton equilibrium of a classical glass electrode. Both sensors exhibit excellent performance in sensitivity, selectivity, and stability, with both pH and NH_4_^+^ responses obeying Nernst slopes, with slopes of 63.8 mV pH^−1^ (pH sensor) and 55.7 mV dec^−1^ (NH_4_^+^ sensor), respectively. The LOD for NH_4_^+^ is 10^−4.8^ mol L^−1^. Furthermore, the potential advantage of the group’s SIM-free design not only greatly reduces costs but also exhibits excellent biocompatibility, providing a possible method for developing the next generation of practical wearable ion sensors.

### 4.2. EDL Capacitance-Type SC-ISEs

**Figure 7:**
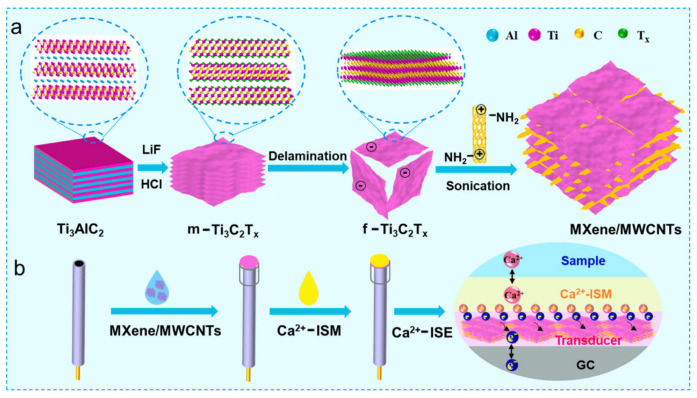
The illustrations for (**a**) the preparation of MXene/MWCNT composites and (**b**) the fabrication of SC-ISE for Ca^2+^ detection [68].

In recent years, MXenes, a class of two-dimensional (2D) transition metal carbides and nitrides [65], have attracted significant attention due to their high specific surface area, large double-layer capacitance, and high metallic conductivity [66,67,68]. These competitive advantages make MXenes a promising SC material for SC-ISEs with great potential. However, due to the hydrophilic groups at the ends of MXenes, such as −OH, −O, and −F [69], it is not conducive to eliminating the water layer between the conductive substrate and the ISM interface. Therefore, introducing a modified material with excellent conductivity and hydrophobicity into MXenes is a necessary condition for preparing stable SC-ISEs. To address this issue, Liu et al. [70] used cationic amino-functionalized multi-walled carbon nanotubes (MWCNTs) as interlayer spacers and introduced negatively charged MXenes via an electrostatic self-assembly method to prepare MXene/MWCNT composite materials as the SC for SC-ISEs (Figure 7), using N, N-dicyclohexyl-2-[2-(dicyclohexylamino)-2-oxoethoxy] acetamide (ETH 129), NaTFPB, PVC, and o-NPOE as membrane components to construct SC-Ca^2+^-ISEs. The study showed that the electrostatic self-assembly method used in this strategy can effectively prevent the aggregation of MXene nanosheets and promote charge transfer, significantly improving DEL capacitance and preventing the formation of a water layer at the interface. This is attributed to the large specific surface area, good chemical stability, and hydrophobicity of MWCNTs. Furthermore, the capacitance of GC/MXenes/MWCNTs/Ca^2+^-ISE (210 μF) was significantly higher than that of GC/MXenes/Ca^2+^-ISE (189 μF) and GC/MWCNTs/Ca^2+^-ISE (67 μF). Under optimal conditions, the constructed sensor achieved an LOD of 7.9 × 10^−7^ M for Ca^2+^ and a Nernst slope of 25.8 ± 0.5 mV/decade. Therefore, MXene/MWCNTs is expected to become a promising SC material.

Graphene has the characteristics of a large specific surface area, strong electron transfer capability, and high chemical stability, and is a two-dimensional carbon nanomaterial. Its unique sp2-hybridized carbon sheet structure at the atomic level endows it with excellent hydrophobicity, which may hinder the formation of water layers and thus provide enhanced stability for the sensor over a longer period [71]. Magdy et al. [72] prepared graphene–PVC nanocomposite materials using a solution dispersion method [73]. Then, the polished GC surface was covered with an SIM (containing graphene–PVC nanocomposite, tetradodecylammonium bromide, and 2-nitrophenyl octyl ether), and an SC-ISE for measuring the level of perindopril (PER) in human plasma was constructed. In this sensor, the addition of graphene–PVC nanocomposite increased the electrode’s surface area and provided additional active sites for ion exchange. Moreover, the superior conductivity of graphene offers an effective pathway for ion transport. The constructed sensor exhibited high sensitivity and selectivity. Under optimal conditions, the LOD of the SC-ISE was 3.2 × 10^−9^ M, with a Nernst slope of 58.89 mV/decade and a response time of 20 s. The analytical GREEnness metric (AGREE) and whiteness (RGB) are the current two metrics used to measure green and sustainable analytical processes. It is noteworthy that the overall AGREE score of this strategy was 0.93, confirming that the method is green and provides a promising design approach to meet the needs of green analytical chemistry (GAC).

Although the sensors proposed in these studies on graphene as the transducer material for SC-ISEs have shown quite satisfactory performance, the irreversible recondensation and aggregation of graphene due to strong interlayer interactions and van der Waals forces have led to a reduction in surface area and electron transfer pathways. In recent years, metal nanomaterials have received increasing attention as SCs for SC-ISEs due to their high conductivity and large specific surface area [74,75,76]. Nguyen et al. [77] prepared gold nanoparticle-reduced graphene oxide (AuNP-rGO) composites using a simple and environmentally friendly one-pot method with sodium citrate as the reducing and stabilizing agent. Subsequently, the nanocomposite was formed as an SC layer on a GC substrate by drop casting. Finally, a layer of an SIM with non-muscle actin as the ion carrier was covered to develop an efficient SC-NH_4_^+^-ISEs based on AuNP-rGO. As expected, the XRD pattern of AuNP-rGO showed a graphitic peak due to rGO stacking near 2θ = 25.38° (002), but the rGO sheets modified with AuNPs exhibited higher electrocatalytic activity than pure rGO, indicating that the hybridization of AuNPs is beneficial for addressing the reduction in surface area and electron transfer pathways caused by rGO stacking. Moreover, the AuNP-rGO layer’s large surface area, high capacitance, and good hydrophobicity effectively enhanced the electrode’s potential stability without forming a water layer. Under optimal conditions, the electrode achieved an LOD of 3.80 × 10^−6^ M for NH_4_^+^, with a good Nernstian response (slope of 56.94 ± 1.57 mV/decade) and rapid potential response (<10 s). Additionally, the electrode exhibited good reproducibility, long lifespan, and insensitivity to interference from light, oxygen, carbon dioxide, and redox substances. This strategy provides a viable alternative for developing durable and efficient SC-ISEs.

**Figure 8:**
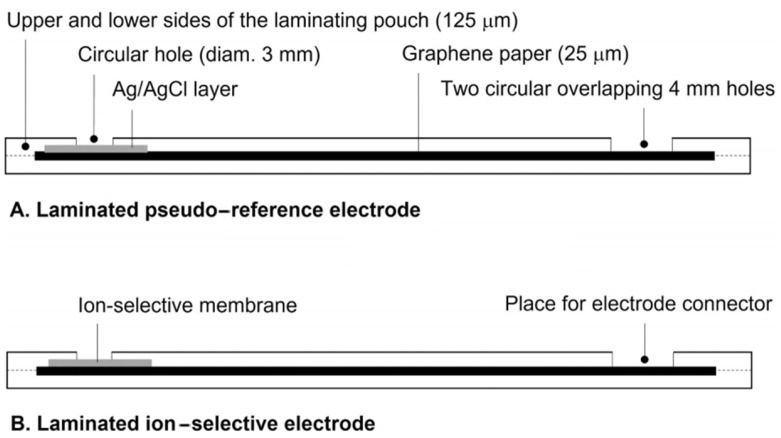
Schematic side view of the construction of the flexible graphene paper-based (**A**) laminated Ag/AgCl pseudo-reference electrode and (**B**) laminated K^+^-selective electrode [83].

In recent years, paper-based SC-ISEs have been widely used in environmental monitoring and medical diagnostics [78]. In sensor design, selecting an appropriate high-selectivity sensing material is crucial. Synthetic molecularly imprinted polymers (MIPs), as receptors targeting target molecules, have high specificity and stability and unique physical, chemical, and mechanical properties, as well as low cost and simplicity in preparation [79]. Hassan et al. [80] developed a solid-state MIP paper potentiometric sensor for monitoring a losartan potassium (LOS-K) drug. First, two rectangular Whatman^®^ filter paper strips with circular carbon ink (diameter 4 mm) were used as platforms (20 × 5 mm each, 180 μm thick). One carbon ink was modified with rGO and coated with MIPs (using LOS-K drug molecule as the template, acrylamide (AAm) as the monomer, ethylene glycol dimethacrylate cross-linker (EGDMA) as the cross-linker) as the recognition electrode, and a solid polyvinyl butyral (PVB) was applied on the second carbon dot as the RE. The formation of hydrogen bonds between highly electronegative atoms in MIPs and the aromatic ring π electron cloud of LOS-K and the interaction between the amide carbonyl of AAm and the C-Cl group of the losartan drug resulted in a high selectivity of the constructed sensor. Moreover, the improvement of the sensing membrane interface EDL capacitance by rGO and the enhancement of the hydrophobic performance of the conductive carbon ink contributed to the enhancement of the sensor’s potential stability (±0.2 mV) and long lifespan (up to 3 months) [81]. Under optimal conditions, the sensor achieved an LOD of 2.7 ± 0.3 × 10^−7^ M for LOS-K with a Nernst slope of −58.2 ± 0.3 mV/decade. It showed good selectivity for acetaminophen, ascorbic acid, dextromethorphan, and other related compounds commonly used by patients with COVID-19. It can be used for rapid medical diagnosis, drug formulation, and losartan determination in human urine samples in patients with COVID-19, as well as for rapid diagnostics in hospitals for overdose patients and quality control/quality assurance testing in the pharmaceutical industry. It is noteworthy that the dual-electrode design of the potentiometric sensor requires a reliable RE to ensure stable reference potentials for sensitive ion detection in SC-ISEs [82]. To ensure that SC-ISEs are matched to a reliable RE, Mandjoukov et al. [83] integrated the RE and SC-ISEs into a single measurement unit to develop SC-K^+^-ISEs with high sensitivity and stability. Initially, a low-cost flexible graphene paper with pre-deposited Ag/AgCl and K^+^-ISM was prepared by lamination, then placed in a lamination bag and sent to a laminator. Unlike previous SC-ISEs, the ISM of this sensor was directly coated on the surface of graphene, thus serving both as an electrode substrate and as an ion–electron transducer (Figure 8). Under optimal conditions, the constructed sensor exhibited good reproducibility in detecting K^+^ in samples, and the Nernst slope and LOD are 57.4 ± 0.3 mV/pK (n = 4) and 6 × 10^−7^ M, respectively. This superior performance is attributed to the simultaneous pressing of RE and K^+^-ISM in a one-step molding process, which not only ensured that the electrode matched a reliable RE to enhance sensitivity but also eliminated the manual drop-casting steps. This not only avoided potential electrode surface defects and reproducibility issues caused by manual operations but also simplified electrode structure and manufacturing processes for mass production [84]. Additionally, the SC-ISEs exhibit minimal photosensitivity and a low cost (<EUR 1), making them potentially useful in blood analysis with relatively constant chloride content.

Similarly, various printing techniques, including screen printing and 3D printing, have been used to simplify carbon-based SC-ISEs. For point-of-care (POC) home testing, there is a need to develop low-cost, portable sensing devices that can be automatically calibrated or require no calibration and can perform frequent POC analyses for untrained users. Electrolytes such as Na^+^, K^+^, and Ca^2+^ in biological fluids play a vital role in maintaining life activities [85]. Currently, for the analysis of electrolytes in human urine, samples require a brief freezing treatment. However, immediate in situ analysis of urine samples can provide the most accurate results. Teekayupak et al. [86] proposed a fully automated system for the mass production of potentiometric sensors based on laser-induced graphene electrodes (LIGEs), which are integrated with a portable radio potentiometer for detecting K^+^, Na^+^, and Ca^2+^ ion contents in real urine and simulated sweat samples. Initially, CB carbon-based materials and Na^+^-SIM (comprising Na ion carrier X, NaTFPB, DOS, and PVC), K^+^-SIM (comprising Valinomycin, KTCIPB, DOS, and high-molecular-weight PVC), and Ca^2+^-SIM (comprising calcium ion carrier II (ETH129), KTCIPB, NPOE, and PVC) were prepared. Then, a computer-controlled semiconductor diode laser scribing micromachining system (a laser-scribing micromachining system with a semiconductor diode laser-controlled by a computer) was used to laser scribe polyimide (PI) into combustion and carbonization to serve as the LIGE substrate. The 3D printing robot was operated to sequentially drop CB carbon-based materials and Na^+^-SIM, K^+^-SIM, and Ca^2+^-SIM onto the LIGE substrate. Finally, a microfluidic device was prepared by laser cutting with PET double-sided tape (thickness 100 μm) and a transparent film (thickness 100 μm) to form channels. The SC-ISEs and PET double-sided tape and transparent film were assembled sequentially to construct an ISE based on the microfluidic device. This device can be directly attached to the human skin, and sweat samples can be collected through nine sample inlet holes (4 mm) and directly flowed to a detection area of 15 mm diameter for potentiometric determination of K^+^, Na^+^, and Ca^2+^ ions. FE-SEM images showed that the laser-scribed PI, after combustion and carbonization, formed a porous multilayer graphene structure with a parallel arrangement on the substrate surface. The group also prepared two other substrates, one where TC303 graphite and carbon ink were applied to a PET substrate using an internal template printing method, and another where PVC substrates were screen-printed with carbon ink. These were compared with the LIGE substrate. The experimental results showed that SC-ISEs prepared with the PET and PI substrates had slopes closer to the theoretical Nernstian values, due to the highly hydrophobic nature of these substrates, which prevented the formation of a water layer at the ISM interface. Moreover, the reproducibility of SC-ISEs fabricated on the PET substrate through internal template printing was slightly better than that of LIGEs, but LIGEs had the advantage of being easily mass-produced through automated systems. The group also compared SC layers made from eight different carbon-based materials, excluding CB, such as MWCNTs and GR. The results indicated that SC-ISEs modified with GR had lower sensitivity, while CB exhibited high sensitivity comparable to MWCNTs. Furthermore, CB had a lower cost than other carbon-based materials, including MWCNTs. Moreover, CB not only showed higher uniformity and hydrophobicity on the modified electrodes, but also increased the electrode’s surface area. Additionally, the potential drift of electrodes modified with CB was significantly lower than that of unmodified electrodes, which can be explained by the conductivity of the SC material. Under optimal conditions, this sensor achieved detection limits of 10^−5^ M for Ca^2+^ with a sensitivity of 30.1 mV/decade^−1^; 10^−5^ M for Na^+^ with a sensitivity of 42.9 mV/decade^−1^; and 10^−4^ M for K^+^ with a sensitivity of 38.03 mV/decade^−1^, consistent with ICP-OES results. This strategy provides a low-cost detection and sensing platform for POC applications.

## 5. Conclusions and Future Perspectives

For ease of comparison, we have compiled the samples, objects, and detection results of the various SC-ISE tests mentioned above in Table 1. Current research focuses on optimizing the assembly components of SIMs, modifying the SC layer, synthesizing new ion–electron transducers, and selecting rational conductive substrates for pre-treatment strategies to construct SC-ISEs with fast response, high selectivity, stability, and sensitivity. However, most research focuses on SC-ISEs with the ISM, neglecting the high cost and the challenging elimination of the impact on potential stability. Therefore, membrane-free SC-ISEs (ISM-free SC-ISEs) are expected to emerge in the field of sensor research due to their advantages of high performance, low cost, long lifespan, lack of water layer effects, and ease of miniaturization. The development of ISM-free SC-ISEs is still in its infancy. The types and quantities of bifunctional materials for constructing ISM-free SC-ISEs are insufficient, and systematic research on their selectivity and response mechanisms has not yet formed, resulting in a lack of theoretical support during the research process. The future development of ISM-free SC-ISEs will mainly focus on the following aspects: (1) Developing simpler and more efficient research strategies to enhance the sensing performance of ISM-free SC-ISEs; (2) Identifying more bifunctional materials that exhibit both ion recognition and ion–electron transduction; (3) Conducting in-depth research on the selectivity and response mechanisms of ISM-free SC-ISEs. Providing theoretical support for membrane-free SC-ISEs in practical applications. With the advent of the Internet of Things and the era of artificial intelligence, solid-state potentiometric sensors, as the front-end sensing devices for digital intelligence, will have greater development potential.

## Figures and Tables

**Table 1:** The samples, objects, and detection results of the various SC-ISEs mentioned above.

The Type of SC-ISEs	Sample Matrix	Ion	Range (mol/L)	Response Slope (mV/Decade)	LOD (mol/L)	Ref
Oxidation–Reduction	Seawater	Pb^2+^	1.0 × 10^−3^ to 1.0 × 10^−6^	28.5 ± 0.8	1.9 × 10^−7^	[38]
	Sediment of Lake	Pb^2+^	1.0 × 10^−7^ to 1.0 × 10^−2^	29.89	1 × 10^−7^	[48]
	Seawater	CO_3_^2−^	2.6 × 10^−5^ to 5.3 × 10^−4^	28.3 ± 0.4	1.1 × 10^−5^	[50]
	Solution (CaCl_2_)	Ca^2+^	1.0 × 10^−6^ to 2.9 × 10^−2^	27.5 ± 0.2	5.0 × 10^−7^	[52]
	Soil	NO_3_^−^	1.0 × 10^−7^ to 1.0 × 10^−1^	58.85	2.5 × 10^−6^	[58]
	Human Plasma	Vildagliptin	1.0 × 10^−8^ to 1.0 × 10^−2^	60.04 ± 1.4	4.5 × 10^−9^	[59]
	Human Biofluids	NH_4_^+^	1.0 × 10^−5^ to 1.0 × 10^−1^	55.7	10^−4.8^	[62]
EDL Capacitance	Solution (CaCl_2_)	Ca^2+^	1.0 × 10^−5^ to 1.0 × 10^−1^	25.8 ± 0.5	7.9 × 10^−7^	[70]
	Human Plasma	PER	5.5 × 10^−8^ to 1.4 × 10^−7^	58.89	3.2 × 10^−9^	[72]
	Lake Water	NH_4_^+^	1.0 × 10^−5^ to 1.0 × 10^−2^	56.94 ± 1.57	3.80 × 10^−6^	[77]
	Human Urine	LOS-K	8. × 10^−5^ to 6.9 × 10^−2^	58.2 ± 0.3	2.7 ± 0.3 × 10^−7^	[80]
	Solution (KNO_3_)	K^+^	10^−5.5^ to 10^−1^	57.4 ± 0.3	6 × 10^−7^	[83]
	Human Urine	Ca^2+^	10^−4^ to 10^−1^	30.1	10^−5^	[86]
		Na^+^	10^−4^ to 10^−1^	42.9	10^−5^	
		K^+^	10^−3^ to 10^−1^	38.03	10^−4^	

## Data Availability

No new data were created or analyzed in this study. Data sharing is not applicable to this article.
